# Sir William Henry Broadbent

**Published:** 1907-08

**Authors:** 


Sir William Henry Broadbent died in London, July 10, 1907, in the seventy-third year of his age. Sir William has been prominent in English medical affairs for many years and has been president of many medical organisations.

In 1892 he was appointed physician in ordinary to the Prince of Wales, after attending the Duke of York through his typhoid fever attack in 1891. He was also in attendance on the Duke of Clarence during his fatal illness in 1892. The following year Dr. Broadbent was created a baronet, and in 1898, on the death of Sir Richard Quain, he received an appointment as physician extraordinary to Queen Victoria. Upon the accession of King Edward, Sir William was continued as physician in ordinary to his majesty and to the new Prince of Wales. He was a Knight Commander of the Victorian Order, a Commander of the Legion of Honor and a Fellow of the Royal Society. He held the degree of LL.D. from both St. Andrew's and Edinburgh universities, and that of D.S. from Leeds University, and was the author of volumes on "The Pulse" (1890) and on "The Heart" (1897).

---

The Treatment of Malaria as Found in New York City.—William Stump of New York, advocates the treatment of malaria by large doses of quinine given just before the expected chill so as to reach the blood at the time when the spores are thrown into the circulation and are in their most susceptible state. He uses quinine in solution, five grains for each fifty pounds of body weight, one hour before the chill, and follows with the same dose in chronological sequence.—*Medical Record,* July 20, 1907.

